# Development of a non-contact sleep monitoring system for children

**DOI:** 10.3389/fdgth.2022.877234

**Published:** 2022-08-08

**Authors:** Masamitsu Kamon, Shima Okada, Masafumi Furuta, Koki Yoshida

**Affiliations:** ^1^ Department of Robotics, Ritsumeikan University, Shiga, Japan; ^2^ Technology Research Laboratory, Shimadzu Corporation, Kyoto, Japan

**Keywords:** sleep stage, sleep monitoring, children, video monitoring, video image processing, machine leaning

## Abstract

Daily monitoring is important, even for healthy children, because sleep plays a critical role in their development and growth. Polysomnography is necessary for sleep monitoring. However, measuring sleep requires specialized equipment and knowledge and is difficult to do at home. In recent years, smartwatches and other devices have been developed to easily measure sleep. However, they cannot measure children's sleep, and contact devices may disturb their sleep.

A non-contact method of measuring sleep is the use of video during sleep. This is most suitable for the daily monitoring of children’s sleep, as it is simple and inexpensive. However, the algorithms have been developed only based on adult sleep, whereas children’s sleep is known to differ considerably from that of adults.

For this reason, we conducted a non-contact estimation of sleep stages for children using video. The participants were children between the ages of 0–6 years old. We estimated the four stages of sleep using the body movement information calculated from the videos recorded. Six parameters were calculated from body movement information. As children’s sleep is known to change significantly as they grow, estimation was divided into two groups (0–2 and 3–6 years).

The results show average estimation accuracies of 46.7 ± 6.6 and 49.0 ± 4.8% and kappa coefficients of 0.24 ± 0.11 and 0.28 ± 0.06 in the age groups of 0–2 and 3–6 years, respectively. This performance is comparable to or better than that reported in previous adult studies.

## Introduction

Sleep is an important part of daily life as it reduces stress and aids recovery. Accordingly, monitoring sleep at home can contribute to health management (1, 2). In addition, sleep plays an important role in the development of children (3) and is also related to their parents’ health (4). Sleep quality in children can contribute to health management. Therefore, it is important to monitor sleep quality in children on a daily basis to detect sleep problems. Polysomnography (PSG) is commonly used to assess sleep quality in clinical practice, but it requires measuring various biological signals, such as electroencephalograms (EEGs), electromyograms, and electrooculograms and requires specialized equipment and knowledge (5–7). Therefore, PSG scoring is not a realistic method for assessing the sleep stage in the home environment. In addition, the installation of various devices may diminish the quality of sleep in children. Thus, measurement methods that reduce the burden on equipment installation are needed.

In recent years, electrocardiograms (ECGs), pulse-rate laser Doppler sensors (8, 9), and cameras (10) have been used to quantify sleep quality in homes (11). In particular, camera-based methods are considered the most suitable for monitoring children’s sleep because they are entirely noncontact and easy to install. This method uses the relationship between body movements and sleep stages, and it has been reported that there are significant differences in the frequencies of body movements among sleep stages (12). Nochino et al. (13) used an infrared camera to measure sleep quality in four stages. However, this method is intended for adults and is unsuitable for assessing sleep in children.

Long et al. (14) quantified sleep features and patterns in children by using an infrared camera. In this study, children’s sleep was estimated during both waking and sleeping stages. However, it is believed that the secretion of growth hormone responsible for children’s development occurs during deep sleep (15). In addition, de Goederen et al. (16) used a radar system to estimate the four stages of sleep in children, and achieved an accuracy of 58.0%. However, the radar system is difficult to install and expensive, thus making it difficult to use at home.

There are various sleep measurement devices, and methods based on body movements (17). Two commonly used devices include the smartwatch and smartphone. Chinoy et al. (18) evaluated the smartwatch Fitbit Alta HR. The device provided an estimation accuracy of 90% for the distinction between sleep and wakefulness for adults. Patel et al. (19) used a dedicated application to assess sleep on smartphones. Their results confirmed the lack of a correlation relationship between the sleep stage determined by the application and that determined by PSG.

Therefore, it is necessary to develop a simple method for monitoring children’s sleep. We used an infrared camera, which is easy to install and is inexpensive, to estimate the four sleep stages of children aged 0–6 years. As it is known that sleep in children changes considerably as they grow, we estimated the sleep stages of children aged 0–2 years and 3–6 years. Previous studies (13) have utilized support vector machines (SVMs) (20). In this study, the extremely randomized trees (Extra Trees) ensemble machine learning algorithm was used.

## Methods

**Figure 1:**
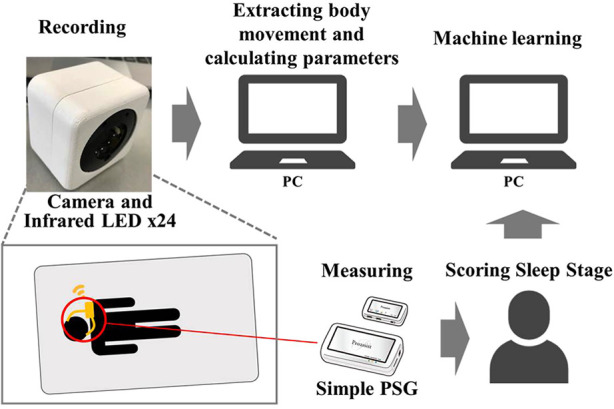
Measurement and analysis methods. A sleeping child was recorded by an infrared camera, and body movements were extracted from the video data. Some features were calculated by the body movements as the training parameter for machine learning. And machine learning was performed using the calculated futures and sleep stage obtained by a simple PSG as the correct data.

In this study, we extracted body movement during sleep from video recordings. Figure 1 shows the measurement and analysis procedure. The measurements were performed in the subjects’ homes. Eight subjects participated who were aged 0–6 years, with a mean age of 2.3 ± 2.1 years. The measurements were conducted for 1–4 nights per person for a total of 14 nights. The variability in the number of nights per person is attributed to the fact that the measurements were conducted at home, and the measurements were not always accurate.

**Figure 2:**
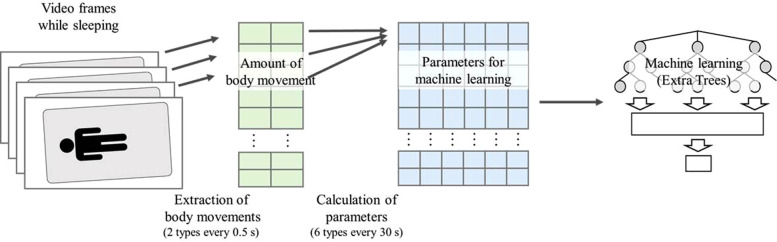
Flow of sleep stage estimation. Two types of body movements were calculated every 0.5 s using difference processing, and six features were calculated every 30 s. Then, machine learning was apply these feature values to sleep stage estimation.

Informed consent for measurements was obtained from the parent of all subjects. The experiment was approved by the Ethical Review Committee for Medical Research Involving Human Subjects, Ritsumeikan University (BKC-Human Medicine-2020-053). A simple PSG was used as the gold standard for learning and evaluating the classifier. This reduced the burden on the child because the sleep stages were assessed with far fewer electrodes than those used in PSG in clinical practice. A small sensor was attached to the body for recordings with the simplified PSG. The recording device was connected wirelessly to reduce the burden on the child during sleep. A simple PSG was used offline by a technician to score the sleep stages. A video of the child sleeping was recorded at the same time as the simple PSG recording. Figure 2 shows the flow of sleep stage estimation from video recordings. Body movements were extracted from the videos using video processing. Six machine learning parameters were calculated from the extracted body movements. Sleep stages were estimated from six parameters using Extra Trees machine learning with the simple PSG sleep stage as the answer response sleep stages.

### PSG recording and sleep stage scoring

Simple PSG scoring was performed using a ZA-X EEG sensor (Proassist, Japan). This device has four electrodes and can measure data to estimate the sleep stage. The results of this simple PSG instrument have been reported to have a concordance rate of 85.5% compared with PSG scoring (21). The measured data were used to estimate the sleep stages automatically every 30 s based on the American Academy of Sleep Medicine (8). Subsequently, a specialist technician corrected the results. Stage W is defined as Wake, stage R as rapid eye movement (REM), stages N1 and N2 as Light, and stage N3 as Deep.

### Region-of-interest selection and body movement extraction

An ELP-USBFHD05MT-DL36-J camera (Shenzhen Ailipu Technology Co., Ltd., China) equipped with infrared light-emitting diodes (LEDs) was used. The camera’s resolution was 1,920 × 1,080 pixels, and measurements were performed at 2 frames per second. The camera was controlled by a Jetson Nano (NVIDIA Corporation, United States of America). The camera was placed around the bedding, so that the entire bedding area could be observed. The camera was placed at a height of about 1–2 m from the bed. For the experimental design, it is better to install the cameras at the same height under the same conditions. However, since this study was conducted under the conditions that each household could install the camera, it was not possible to standardize the height conditions.

**Figure 3:**
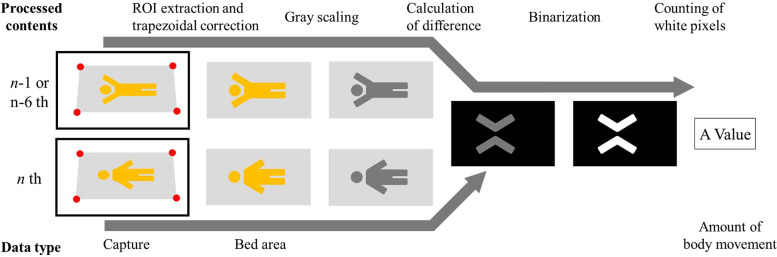
Procedure of the body movement extraction. Only the bed area was extracted from the video using trapezoidal correction, and converted to a black-and-white image using grayscaling, differencing, and binarization processes. The number of white color pixels, It means that a pixel has had a change in luminance value since the previous frame, was counted as the amount of body movement.

Figure 3 shows the body movement extraction procedure. The bedding was extracted as a region-of-interest (ROI) in the video for body motion extraction. The ROI extraction eliminated body movements (other than those related to the subject), such as the unintended appearance of parents. The ROI was set manually by the researcher. The extracted ROIs were transformed by trapezoidal correction according to their size. For the extraction of body motion, we used differences and binarization. The difference calculation method is shown in Equation 1. $I_{d}$ is the difference image, $I_{n}$ is the n th frame, *x* is the vertical pixel coordinate, and *y* is the horizontal pixel coordinate.

(1) $$\begin{array}{c} I_{d}(x,y)=|I_{n}(x,y)-I_{n-1}(x,y)| \end{array}$$

In this process, the difference in pixel-by-pixel luminance values between the *n*th and the (*n* − 1)th frames of the grayscale video was calculated. In the binarization process, pixels above the threshold were converted to white pixels (luminance value: 255), and the pixels below the threshold to black (luminance value: 0). Finally, the number of white pixels was counted as the amount of body movement. Equation 2 shows the equation for the amount of body motion. $dif\;f_{n}$ is the amount of body motion in the nth frame, $I_{bn}$ is the image after binarization in the nth frame *x* is the vertical pixel coordinate, and *y* is the horizontal pixel coordinate.

(2) $$\begin{array}{c} dif\;f_{n}=\frac{\sum_{i=1}^{x}\sum_{\;j=1}^{y}I_{bn}(x,y)}{255} \end{array}$$

Motion extraction was performed twice, every 0.5 s, and two motion values were extracted. The first amount of body movement was calculated between the *n*th and (*n* − 1)th frames, corresponding to a very small temporal difference (0.5 s), allowing us to detect fast body movements. The second amount of body movement was computed between the *n*th and (*n* − 6)th frames, which corresponds to a large temporal difference (3.0 s), therefore allowing the detection of slow body movements.

### Sleep stage estimation

Extra Trees was used to estimate the sleep stages from body movements (22). To use this classification method, six parameters were calculated from two types of extracted body movements. These parameters were calculated once every 30 s. This process is the same timing as the determination of sleep stage based on simple PSG scoring. Extra Trees was then trained using six parameters and the sleep stages from simple PSG scoring. Sleep stages were estimated sequentially by using the six parameters. The amount of body movement varied with sleep stages (12). The amount of physical activity was related to sleep, even in children (23, 24). In Wake, body movements were large and frequent; in Light and REM, body movements tended to be more frequent, but the number of body movements was greater in Light than in REM. Conversely, in the case of Deep, body movements were almost absent. These results indicate that the average amount of body movements, frequency of body movements, and elapsed time are necessary to identify sleep stages. Therefore, six parameters were calculated using the following equation to represent the characteristics necessary for estimation. Note that $dif{\;f}_{i,0.5}$ is the fast body movement of the *i* th frame, and $dif{\;f}_{i,3.0}$ is the slow body movement of the *i* th frame.

Parameter 1: The first parameter was estimated using Equation 3 (n=1,61,121,⋯). This parameter is a moving average of body movements during 30 s and represents the magnitude of body movements. This parameter was used for Wake classification.

(3) $$\begin{array}{c} SumMean_{30}=\frac{\sum_{i=n}^{n+59}dif{\;f}_{i,0.5}+dif{\;f}_{i,3.0}}{60} \end{array}$$

Parameter 2: The second parameter was estimated based on Equation 4 (n=1,61,121,⋯). This parameter is the logarithm of the moving average over a period of 300 s. If the result is less than one, then it is replaced by zero. If it is greater than one, then the logarithm is calculated. This parameter represents the frequency of body movements over 5 min and is used to discriminate between REM and Light.

(4) $$\begin{array}{c} PropMean_{300}=\log_{10}\left(\frac{\sum_{i=n}^{n+599}dif{\;f}_{i,0.5}\times dif{\;f}_{i,3.0}}{600}\right) \end{array}$$

Parameter 3: The third parameter is given by Equation 5 (n=1,61,121,⋯). This parameter is a moving average over a period of 300 s and represents the magnitude of body movement over a long period of time. This parameter was used to discriminate the long-time Wake and long-time Deep with little movement.

(5) $$\begin{array}{c} SumMean_{300}=\frac{\sum_{i=n}^{n+599}(dif{\;f}_{i,0.5}+dif{\;f}_{i,3.0})}{600} \end{array}$$

Parameter 4: The fourth parameter is given by Equation 6. This parameter is the duration of frames during which body motion is below a certain level, thus representing the duration of the stationary state. Similar to parameter 3, this parameter was used to discriminate between Deep and little movements.

(6) $$\{\begin{array}{rl} & NonBM=0\;dif{\;f}_{i,0.5}+dif{\;f}_{i,3.0}>k\\ & NonBM=NonBM+1dif{\;f}_{i,0.5}+dif{\;f}_{i,3.0}\leq k \end{array}$$

Parameter 5: The fifth parameter is given by Equation 7 (n=1,61,121,⋯). This is the time elapsed since the start of the measurements. This parameter represents the number of epochs. There was a tendency for deeper sleep in the first half and more REM in the second half. Therefore, this parameter was used to determine the difference between Deep and REM.

(7) $$\begin{array}{c} Epoch=\frac{n+59}{60} \end{array}$$

Parameter 6: The sixth parameter is given by Equation 8 (n=1,61,121,⋯). This parameter denotes the dispersion of body movements during a period of 30 s and represents the frequency of body movements. It was used to classify Deep, which contains few body movements, and REM, which contains many body movements.

(8) $$\begin{array}{r} \begin{array}{c} Variance_{30}=\\ \left(\overset{n+59}{\underset{i=n}{\sum }}{\left(dif{\;f}_{i,0.5}+dif{\;f}_{i,3.0}\right)}^{2}-{\left(\bar{dif{\;f}_{i,0.5}+dif{\;f}_{i,3.0}}\right)}^{2}\right) \end{array} \end{array}$$

The size of a child’s body varies considerably at different stages of growth. In this study, the amount of body movement used varied considerably depending on the size of the child’s body. Sleep duration also varied between the subjects and days. This may lead to inaccurate estimation of the sleep stages. Therefore, all parameters were standardized to have a mean of zero and a variance of one. Standardization was implemented based on Equation 9, where *i* denotes the number of parameters, *Param_i_* is the *i* th parameter, $\bar{Param_{i}}$ is the average of the *i* th parameter, and $S_{Param_{i}}$ denotes the variance of the *i* th parameter.

(9) $$\begin{array}{c} Standard\_Param_{i}=\frac{Param_{i}-\bar{Param_{i}}}{S_{Param_{i}}} \end{array}$$

These parameters constitute discrete data. The parameters were not expected to differ considerably for each sleep stage. Therefore, the classifier needs to be trained by dividing the data into smaller pieces. For this reason, we used Extra Trees based on decision trees as the classifier used in this study.

**Figure 4:**
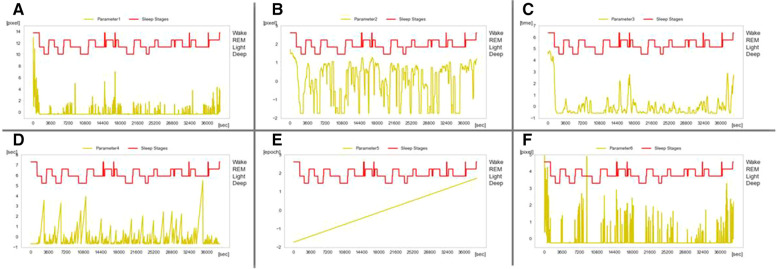
Sleep stage and calculated parameters. (**A**) Moving average over 30 s and represents the magnitude of the body movement over a short period of time. (**B**) Logarithm of the moving average over 300 s and representation of the frequency of body movements over a long period of time. (**C**) Moving average over 300 s and representation of the magnitude of the body movement over a long period of time. (**D**) Duration of body movements below a certain level and representation of the duration of stillness. (**E**) Number of frames converted to the number of epochs and representation of the elapsed time. (**F**) Variance of body movements over a period of 30 s, and representation of the frequency of body movements over a short period of time.

Examples of the results for the six calculated parameters are presented in Figure 4. Because the tendency of the sleep stages varied considerably depending on the child’s development, machine learning with the Extra Trees classifier was used to estimate and evaluate the two groups of children (0–2 years old, and 3–6 years old).

Leave-one-out cross-validation was performed to evaluate the classification performance, and accuracy, sensitivity, and specificity were calculated for each of the four sleep stages to evaluate the classification performance. The overall estimated accuracy and Cohen’s kappa coefficient (25) were calculated.

## Results and discussion

### Results of simple PSG scoring

In total, 50 nights were evaluated: 21 nights for children with ages in the range of 0–2 years, and 29 nights for those in the 3–6 year age range. However, in many cases, scoring with the simple PSG failed, and the data available for analysis were limited to six nights for children aged 0–2 years old, and eight nights for children aged 3–6 years old (a total of 14 nights). Most of the simple PSG scoring failures were caused by children disliking the electrodes and removing them. Another reason was the difficulty in operating the equipment. Although PSG scoring is necessary for research, PSG scoring is not a suitable method for daily monitoring of sleep stages in children.

**Figure 5:**
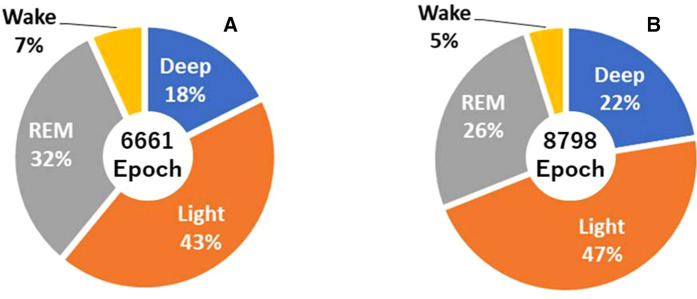
(**A**) Percentages of sleep stages determined by simple polysomnography (PSG) at ages in the range of 0–2 years. (**B**) Percentage of sleep stages determined by simple PSG at ages in the range of 3–6 years.

The percentage of each sleep stage obtained by PSG as correct data is shown in Figure 5. The epochs are the total number of sleep stages determined once every 30 s from PSG. From this, REM sleep is more common in children aged 0–2 years old, while REM is less common in children aged 3–6 years old due to changes in the sleep cycles. However, in clinical PSG examinations, the depth was approximately 30% in normal subjects (26). However, the simple PSG scoring results were low: 18% in children who were 0–2 years old, and 22% in children who were 3–8 years old. This may be because the device used was a simple PSG scoring device, the subjects were children, and their sleep cycles were not stable.

### Sleep stage estimation

**Figure 6:**
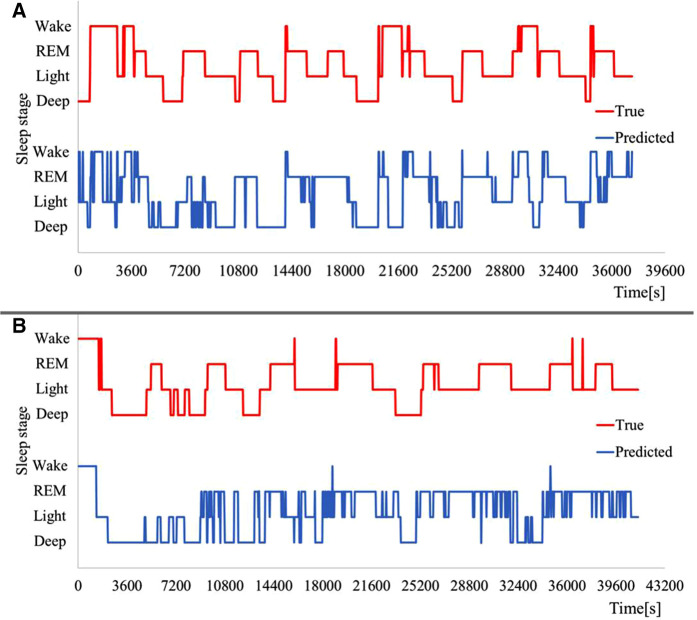
Example of sleep stage estimation results. (**A**) Children with ages in the ranges of (**A**) 0–2 years, and (**B**) 3–6 years.

Examples of estimated sleep stages are shown in Figure 6. Table 1 presents the estimated results for each sleep stage. Table 2 shows the estimation accuracy and kappa coefficient of each dataset. The estimation accuracy of Wake was 90.3 ± 5.1% and was the highest in the age group of 0–2 years, and 97.5 ± 1.3% in the age group of 3–6 years. This was owing to frequent body movements during wakefulness, which was well characterized by parameter 1. However, the specificity of the 4th, 5th, and 6th datasets of children with ages in the range of 0–2 years was low. This may be owing to the short time between bedtime and sleep onset and the low frequency of mid-waking. The same tendency was observed in the second children data with ages in the range of 3–6 years. The estimation accuracy of Light was the lowest in both grooves, and its sensitivity and specificity were also low. This was due to the fact that Light accounts for approximately half of the sleep period and includes the transition period to other sleep stages. Therefore, it is inevitable that misclassification will increase in machine learning. The accuracy of REM was 70.6 ± 7.2% in children with ages in the range of 0–2 years, and 66.8 ± 4.7% in the 3–6 age group, thus indicating that the accuracy of REM estimation was higher in the 0–2 age group compared with that for children the 3–6 age group. This was because REM accounts for a larger proportion of total sleep in children with ages in the range of 0–2 years. In addition, children’s sleep showed the same performance as that of adults in the previous study, despite a higher percentage of REM sleep. This is attributed to the effects of parameters 2, 5, and 6. The accuracy of Deep sleep estimation was 75.9 ± 3.7% in children with ages in the range of 0–2 years, and 79.5 ± 3.3% in children with ages in the range of 3–6 years, probably because parameters 3–6 could be used to determine Deep sleep. However, the low specificity of Deep sleep in children with ages in the range of 0–2 years may be owing to the low number of Deep sleep patterns in the sleep stages as judged by simple PSG. The accuracy of the estimation of each sleep stage is comparable to that reported in a previous study on adults (13).

**Table 1:** Estimation of the different sleep stages for each studied subject.

Age	Stages		Data number								Average
			1	2	3	4	5	6	7	8	
0–2	Deep	Accuracy	73.7	78.1	78.9	73.0	71.5	80.4	–	–	75.9 ± 3.7
		Sensitivity	31.8	66.5	16.3	23.8	58.3	66.4	–	–	43.8 ± 22.5
		Specificity	19.9	37.3	21.5	35.6	37.5	50.4	–	–	33.7 ± 11.4
	Light	Accuracy	60.8	62.9	47.9	49.8	54.8	62.9	–	–	56.5 ± 6.7
		Sensitivity	36.6	34.2	37.3	38.3	26.1	34.4	–	–	34.5 ± 4.4
		Specificity	60.4	53.9	44.0	37.5	51.2	63.1	–	–	51.7 ± 9.7
	REM	Accuracy	79.8	75.6	60.1	70.6	64.9	72.8	–	–	70.6 ± 7.2
		Sensitivity	85.0	70.6	50.4	47.5	61.1	65.4	–	–	63.3 ± 13.8
		Specificity	61.7	59.1	42.2	54.8	43.6	61.9	–	–	53.8 ± 8.9
	Wake	Accuracy	93.3	86.1	95.4	83.2	95.2	88.5	–	–	90.3 ± 5.1
		Sensitivity	65.2	40.8	96.5	44.6	28.6	83.3	–	–	59.8 ± 26.4
		Specificity	75.3	54.0	49.1	14.5	36.4	3.9	–	–	38.9 ± 26.4
3–6	Deep	Accuracy	80.8	82.2	82.6	79.0	73.8	78.4	76.1	84.4	79.5 ± 3.3
		Sensitivity	69.7	71.4	67.6	70.4	54.3	65.5	51.4	75.2	66.5 ± 6.3
		Specificity	52.8	57.4	54.4	45.5	58.7	46.3	58.4	68.8	52.5 ± 5.5
	Light	Accuracy	58.5	57.5	57.2	55.0	51.0	49.4	58.7	50.5	54.8 ± 3.8
		Sensitivity	33.5	45.6	29.2	29.1	34.4	20.5	51.9	16.7	32.0 ± 8.3
		Specificity	62.2	66.7	60.3	52.4	33.1	43.6	45.6	44.3	53.1 ± 12.7
	REM	Accuracy	72.1	71.5	65.1	68.5	63.5	60.2	68.7	58.5	66.8 ± 4.7
		Sensitivity	71.8	53.4	73.9	67.7	27.4	58.9	39.4	67.1	58.8 ± 17.3
		Specificity	45.5	39.9	44.5	49.5	30.0	34.0	41.6	29.1	40.6 ± 7.4
	Wake	Accuracy	97.3	95.1	98.6	98.8	96.5	97.4	99.1	97.2	97.3 ± 1.4
		Sensitivity	89.7	75.0	85.1	74.6	97.8	71.4	91.2	66.7	82.3 ± 10.3
		Specificity	76.1	9.2	78.4	95.7	62.9	89.6	96.3	73.9	68.6 ± 31.2

**Table 2:** Total accuracy and kappa coefficient outcomes.

Age		Data number								
		1	2	3	4	5	6	7	8	Average
0–2	Total accuracy [%]	53.8	51.4	41.2	38.3	43.2	52.3	–	–	46.7 ± 6.6
	Kappa	0.35	0.33	0.1	0.12	0.19	0.32	–	–	0.24 ± 0.11
3–6	Total accuracy [%]	54.36	53.15	51.83	50.66	42.42	42.71	51.3	45.3	49.0 ± 4.8
	Kappa	0.37	0.3	0.31	0.29	0.18	0.21	0.3	0.25	0.28 ± 0.06

Table 2 shows that the mean accuracy of the estimates for the age group 0–2 years old is 46.7 ± 6.6%, and the kappa coefficient is 0.24 ± 0.11. In the age group of 3–6 years old, the mean accuracy of the estimation is 49.0 ± 4.8%, and the kappa coefficient is 0.28 ± 0.06. In a previous study (13), the results for adults yielded an average estimation accuracy of 40.5 ± 2.2% and a kappa coefficient of 0.19 ± 0.04, which are considered to reflect an equivalent or a better performance. We believe that this is caused by the normalization of the parameters. This may have reduced the effects of differences in body size and total sleep time.

**Figure 7:**
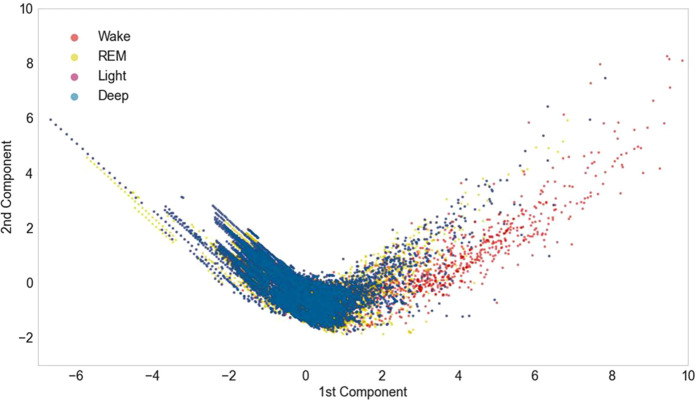
Principal component analysis (PCA) results for six parameters, namely, magnitude of short-time body movement, frequency of long-time body movement, magnitude of long-time body movement, rest time, elapsed time, and frequency of short-time body movement. The horizontal axis is the first principal component, and the vertical axis is the second principal component.

Another reason for the improved accuracy is attributed to the fact that we changed the classifier from an SVM to Extra Trees. Figure 7 shows the results of the principal component analysis (PCA) for the six parameters used for visualization (27). The repeatability of the PCA was 66.8%. The results show that Extra Trees is more suitable than SVM (which uses boundaries) because the difference between the features of each stage is very small in the sleep stage.

Liang et al. (28) proposed a method to estimate sleep stages using Fitbit. In this study, they reported that the estimation accuracy of the four sleep stages for adults was 73.1 ± 11.9%. Although the accuracy of our method was lower than that of the study by Liang et al. (28), it is sufficient to be able to conduct complete measurements without contact, and to be applicable to children. In this study, a simple PSG was used as the reference for actual sleep stages. However, even the clinically used PSG tests are not completely consistent among specialist technicians (29, 30).

The estimated results for the age group of 0–2 years were lower than those for the age group of 3–6 years. This is because the sleep cycle of children with ages in the range of 0–2 years is less stable than that of children with ages in the range of 3–6 years. This is also confirmed by the variance, which shows that the estimation accuracy and variance of the kappa coefficient are greater for children with ages in the range of 0–2 years.

Although our system is less accurate than contact devices, it is useful because it can be used for children with ages in the range of 0–6 and because it is noncontact. However, our system also has limitations. The system does not measure autonomic nervous system activity. Thus, these indicators are also needed if high-estimation accuracy is desired. Therefore, it is possible to reduce the misclassification of Deep and REM to Light.

Extra Trees was used in this study. However, as there are many types of classifiers, it is necessary to compare the performance with other classifiers.

## Conclusions

The estimation of sleep stages in children has not been studied as comprehensively as studied in adults owing to difficulties in measurement and PSG scoring. In this study, we developed a sleep monitoring system for children with the use of an infrared camera. The developed system extracted body movements from a video and calculated six parameters from body movements. The system then used Extra Trees to estimate the four stages of sleep from the parameters. The accuracy of the developed system was approximately 45% on average compared with that of the simple PSG. However, the average estimation accuracy for Deep, where growth hormone is said to be secreted, was more than 75%. The performance of this system was comparable to or better than those reported in previous studies. For these reasons, it is suggested that this system can be used as a noncontact sleep monitoring system for children at home.

Our system can estimate four sleep stages, but not the REM/NREM sleep cycle. It is known that children’s growth can be confirmed by the REM/NREM sleep cycle [26]. Therefore, developing a method to estimate the REM/NREM sleep cycle is necessary.

## Data Availability

The original contributions presented in the study are included in the article/supplementary material, further inquiries can be directed to the corresponding author/s.
